# Whole mitochondrial genome sequence and phylogenetic relationships of Williams’s jerboa (*Scarturus williamsi*) from Turkey

**DOI:** 10.7717/peerj.9569

**Published:** 2020-07-16

**Authors:** Osman İbiş

**Affiliations:** 1 Department of Agricultural Biotechnology, Faculty of Agriculture, Erciyes University, Kayseri, Turkey; 2 Genome and Stem Cell Center (GENKOK), Erciyes University, Kayseri, Turkey

**Keywords:** Williams’s jerboa, Next-generation sequencing, Mitogenome, Phylogeny, Dipodoidea

## Abstract

Williams’s jerboa (*Scarturus williamsi*), a medium-sized jerboa distributed in Anatolia and its adjacent regions, is a member of the four- and five-toed jerboas found mostly in Asia. Disagreements about the taxonomy of this taxon at the genus/species level continue to exist. Here, we report the first effort to sequence and assemble the mitochondrial genome of Williams’s jerboa from Turkey. The mitochondrial genome of *S. williamsi* was 16,653 bp in total length and contained 13 protein-coding genes (PCGs), 22 transfer RNAs (tRNAs), two ribosomal RNAs (rRNAs), and two non-coding regions (the D-loop and O_L_ region) with intergenic spacer. All of the genes, except *ND6* and eight tRNAs, were encoded on the heavy chain strand, similar to the features of mitogenomes of other rodents. When compared with all available rodent mitochondrial genomes, Williams’s jerboa showed (1) a serine deletion at the 3′-end of the *ATP8* gene, (2) the *ND5* gene terminated with a TAG codon and (3) a tandem repeat cluster (273 bp in length) in the control region. Williams’s jerboa and Siberian jerboa grouped as sister taxa despite the high genetic distance (17.6%) between them, belonging to Allactaginae. This result is consistent with the latest pre-revision, which suggests that Williams’s jerboa and the Siberian jerboa may belong to separate genera, as *Scarturus* and *Orientallactaga*, respectively. The present study provides a reference mitochondrial genome for Williams’s jerboa for further molecular studies of other species of Dipodoidea and Rodentia.

## Introduction

The superfamily Dipodoidea (Myomorpha, Rodentia, Mammalia), a monophyletic group represented by 17 genera and 54 extant species, is widely distributed in the Holarctic region (Holden, Cserkész & Musser, 2017; Whitaker, 2017; Michaux & Shenbrot, 2017). Considering the recent studies (Malaney, Demboski & Cook, 2017; Shenbrot et al., 2017; Lebedev et al., 2018; Bannikova et al., 2019; Cserkész et al., 2019), the number of genera and species could increase to 18 and 71, respectively. Holden & Musser (2005) and Lebedev et al. (2013) stated that the superfamily Dipodoidea comprises of three ecomorphotypes (birch-mice, jumping mice and jerboas) that are traditionally classified within a single-family (Dipodidae) or placed in up to six families. However, the number of ecomorphotypes is larger, and each subfamily of Dipodidae can be considered as a separate ecomorphotype (Michaux & Shenbrot, 2017). The taxonomy and phylogeny of Dipodoidea remain controversial, and the phylogenetic relationships among the main lineages have not been clearly established (Michaux & Shenbrot, 2017). The latest classification (Michaux & Shenbrot, 2017) is followed in the present study because of the lack of a comprehensive revision of Dipodoidea.

The genus *Scarturus*, which includes four- and five-toed jerboas, is typically found in arid/semi-arid areas in Asia and north-eastern Africa. According to the latest taxonomic revision (Michaux & Shenbrot, 2017), this genus is represented by seven species (*S. williamsi, S. aulacotis*, *S. euphraticus*, *S. hotsoni*, *S. elater*, *S. vinogradovi* and *S. tetradactylus*). However, the taxonomy of the genus *Scarturus* and its number of species are still controversial (Lebedev et al., 2013; Hamidi, Darvish & Matin, 2016; Michaux & Shenbrot, 2017; Bannikova et al., 2019). Molecular data are available for only a few areas and species within the genus (Dianat et al., 2010, 2013; Kryštufek et al., 2013; Lebedev et al., 2013; Zhang et al., 2013; Moshtaghi et al., 2016; Bannikova et al., 2019). Although intraspecific variations in the characters used in species delimitation are known to exist, the taxonomy of *Scarturus* has largely been based on morphology (Shenbrot, 2009). The taxonomic status of the genus and species is often evaluated via phylogenetic analyses of molecular markers. Dianat et al. (2013), Kryštufek et al. (2013), Moshtaghi et al. (2016) and Bannikova et al. (2019) suggested that the number of species of *Scarturus* may be greater than seven. Darvish et al. (2008) described *S. toussi* (Toussi jerboa) as a new species from Iran based on morphological data. Morphological (Shenbrot, 2009) and molecular (Dianat et al., 2013) data also indicate that *S. hotsoni* and *S. firouzi* should be synonymized as a single species.

Three morphospecies of the genus *Scarturus*, *S. elater* (small five-toed jerboa), *S. euphraticus* (Euphrates jerboa), and *S. williamsi* (Williams’s jerboa), are distributed in Turkey (Çolak, Kivanç & Yiğit, 1994, 1997a, 1997b; Çolak & Yiğit, 1998a, 1998b; Kryštufek & Vohralík, 2005). The distributional area of *S. williamsi* ranges from Turkey to the Caucasus, Iran and southern Lebanon (Çolak, Kivanç & Yiğit, 1994; Kryštufek et al., 2013; Michaux & Shenbrot, 2017). Few studies have been conducted on the Turkish populations of Williams’s jerboa. Of the available studies, most are based on morphology (Çolak, Kivanç & Yiğit, 1994, 1997a) and karyology (Çolak, Kivanç & Yiğit, 1994, 1997b; Arslan & Zima, 2010; Aşan, Toyran & Albayrak, 2010; Arslan et al., 2012). Only a few studies have used mitochondrial sequences to investigate the Turkish populations of *S. williamsi* (Kryštufek et al., 2013; Olgun Karacan et al., 2019, https://www.ncbi.nlm.nih.gov/nuccore/MG255335.1).

Mitochondrial DNA sequences are useful markers for inferring the phylogenetic relationships of mammals and have been widely used to study the origin, evolution, population genetics and phylogenetic relationships of rodents (Irwin, Kocher & Wilson, 1991). However, a single mitochondrial gene or region may be insufficient to resolve phylogenetic relationships (Steppan & Schenk, 2017). Because of the rich content of phylogenetically informative sites, whole mitochondrial genome (hereinafter referred to as mitogenome) data can provide more reliable and robust results (Yang, Xue & Han, 2013; Yue et al., 2015). The data obtained from mitogenomes in recent years have enabled a better understanding of the phylogenetic relationships within various mammalian families (i.e., Bovidae and Cervidae (Wada et al., 2010), Cricetidae (Ding et al., 2019), Ursidae (Krause et al., 2008), Viverridae (Mitra et al., 2019)). However, the mitogenome data of Dipodoidea species are limited; indeed, data of only a few species (e.g., *Orientallactaga sibirica*, *Dipus sagitta*, *Euchoreutes naso*, *Eozapus setchuanus*, *Jaculus jaculus*, *Sicista concolor*, *Stylodipus telum*) (Yue et al., 2015; Ding et al., 2016; Luo & Liao, 2016; Luo, Ding & Liao, 2016; Reyes et al., 2016, https://www.ncbi.nlm.nih.gov/nuccore/AJ416890.1) are available. Thus far, the complete mitogenome sequence of *S. williamsi* has not been characterized and the taxonomic status and phylogenetic relationships of this species within Dipodoidea remain debated because of the limited molecular data currently available.

To rectify these limitations, the present study aimed to (i) generate and assemble the first mitogenome data of Turkish *S. williamsi* by using the next-generation sequencing platform, (ii) characterize the mitogenome of the species by comparing it with the available mitogenomes of other jerboa species, and (iii) reveal the phylogenetic relationships of *S. williamsi* based on previously published and available mitogenome data in GenBank of Dipodoidea/Myomorpha species.

## Materials and Methods

### Sampling, gDNA extraction and long-range PCR amplification

A tissue sample (muscle) was obtained from one female individual of *S. williamsi* collected from Yeşilli Village, Sulakyurt, Kırıkkale (road-killed individual; collection number: 484). Genomic DNA (gDNA) isolation was conducted using the QIAGEN DNeasy^®^ Blood & Tissue Kit by following the manufacturer’s instructions. The quantity of gDNA was determined by using the Qubit^®^ 2.0 Fluorometer and Qubit^™^ dsDNA BR Kit, and an aliquot of the extract was visualized on a 1% agarose gel. The mitogenome was amplified from gDNA by means of Long-Range PCR with NEB LongAmp^®^ Taq 2× Master Mix (M0287S, NEB) in two overlapping fragments (~11 kb and ~7.1 kb in length) using two specific primer pairs (Table S1). The Long-Range PCR mixture contained 1× NEB LongAmp^®^ Taq 2× Master Mix (12.5 µL), 0.6 μM of each primer (1.5 µL of each 10 μM primer) and ~30 ng gDNA (1 µL). The PCR conditions included a pre-denaturation at 94 °C for 1 min, followed by 30 cycles of denaturation at 94 °C for 30 s, annealing at 57 °C (CrocAL1-2024L-CrocBH1-13002H)–55 °C (ScVu-11712L-LuLu-2503H) for 45 s, and extension at 65 °C for 11 min (CrocAL1-2024L-CrocBH1-13002H)–7.5 min (ScVu-11712L-LuLu-2503H). A final extension step was carried out at 65 °C for 10 min. A negative control (no gDNA) was included in the reaction for to detect possible contamination. A total of 5 µL of the PCR products were run on 1% agarose gel, and 4 µL of the PCR products was used to determine the DNA concentration with the Qubit^™^ dsDNA BR Assay Kit according to the manufacturer’s protocol. The standardized PCR products were diluted with ddH_2_O, and 1 ng of amplicon (5 µL) was taken to prepare the DNA sequencing library.

### Preparation of libraries, sequencing and data analysis

The sequencing library was constructed by using the Nextera XT DNA Library Prep Kit (Illumina, San Diego, CA, USA) and Nextera XT DNA Library Preparation Index Kit v2 Set A (Cat. No: FC-131–2001, Illumina, San Diego, CA, USA) following the protocols of the manufacturer. The quantity of each library was normalized via bead-based normalization. The library loading volume and concentration were determined by using the Qubit^™^ dsDNA HS Kit. Library sequencing was performed by using the MiSeq Reagent Kit v2 (500 cycles) (Illumina) and Illumina MiSeq sequencing platform (Genome and Stem Cell Centre, GENKOK, Erciyes University).

Analysis of the obtained 656,266 raw paired-end (2 × 250 bp) reads (average length, 215.7 bp) was carried out using Geneious Prime^®^ v2019.1.3 (Kearse et al., 2012). We used the BBDuk Trimming Tool in Geneious Prime to filter (remove) the following from the raw-read data: short reads (<50 bp), low-quality bases (*Q*-score <20), and adapters. The remaining 577,554 reads from *S. williamsi* were assembled to the *O. sibirica* mitogenome (National Center for Biotechnology Information (NCBI) accession number: KX058130) using the Geneious Mapper algorithm with the following parameters: Sensitivity; Highest sensitivity/Medium and Fine Tuning; iterate up to 25 times. Contig sequences were obtained, and gene annotations were performed based on the *O. sibirica* mitogenome. Moreover, gene borders were checked by using MITOS2 (Bernt et al., 2013) and manually curated. The Tandem Repeats Finder Web server (Benson, 1999) was used to determine sequence repeat motifs in the control region (D-loop). AT-skew and GC-skew analyses were calculated using the formulas (A − T)/(A + T) and (G − C)/(G + C), respectively. The graphical mitogenome map of Williams’s jerboa was drawn with the CGView Server (Grant & Stothard, 2008).

### Phylogenetic analyses

The *S. williamsi* mitogenome obtained in this study and the available mitogenome data from taxa belonging to the suborder Myomorpha (*n* = 43) in the GenBank database (NCBI) were combined into a multiple sequence alignment using the MAFFT algorithm (Katoh et al., 2002). After the control region was removed from the alignments, a total of 16,169 bp remained for phylogenetic analyses. The most suitable substitution model was determined according to the AICc (corrected Akaike Information Criterion) and BIC (Bayesian Information Criterion) criteria by using jModeltest 2.1.10 (Darriba et al., 2012). The Maximum Likelihood (ML) and Bayesian Inference (BI) methods were used to reconstruct phylogenetic trees. The ML tree was generated with MEGA7 (Kumar, Stecher & Tamura, 2016) using the GTR+G+I model and 10,000 bootstrap pseudo-replicates, while the BI tree was constructed with MrBayes v3.2.6 (Ronquist et al., 2012) with four million generations of Markov Chain Monte Carlo iterations. Genetic distance values were calculated in MEGA7 using the Kimura 2-parameter (K2P) nucleotide substitution model.

## Results and discussion

### Mitogenome characterization of Williams’s jerboa

**Figure 1:**
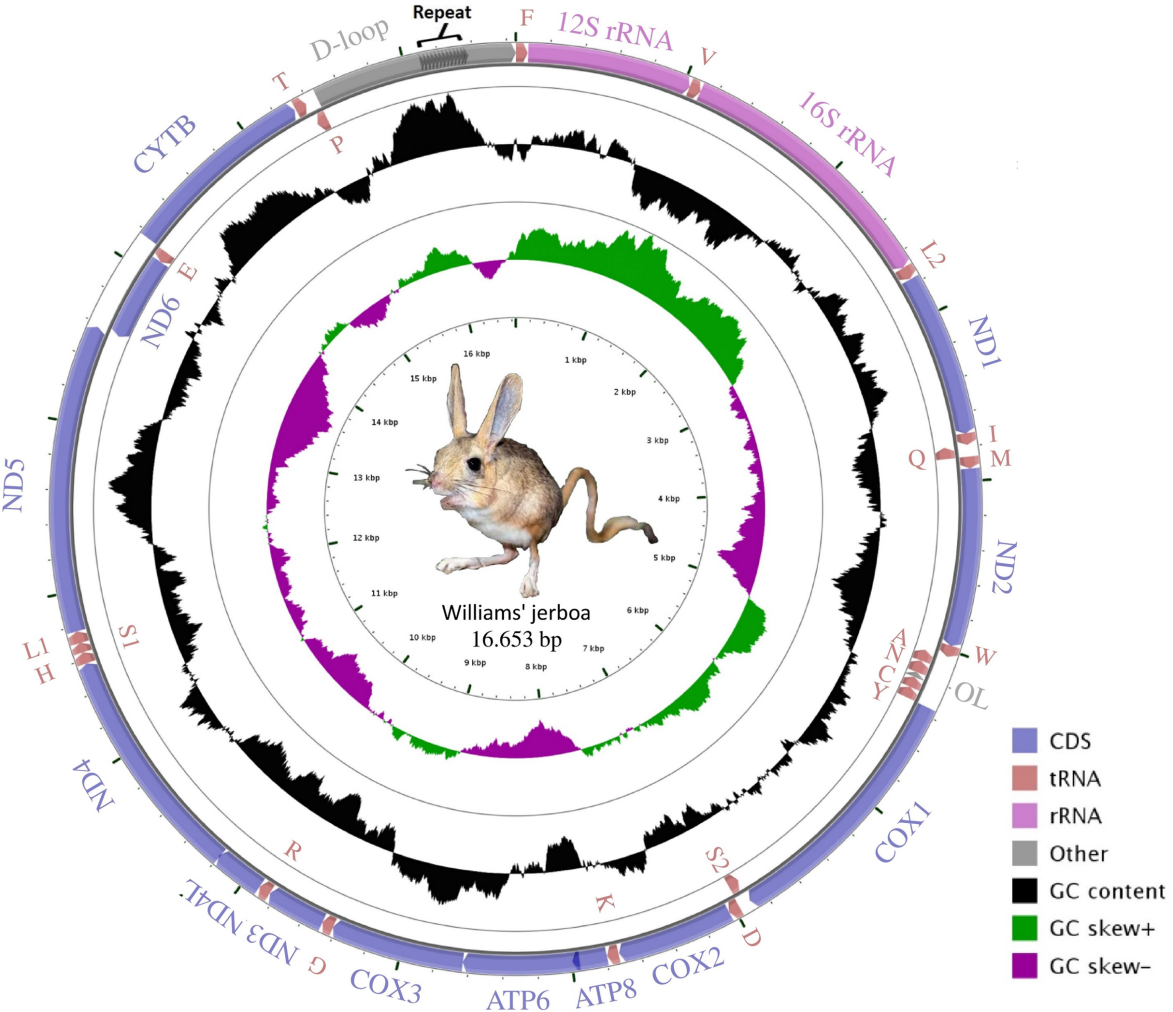
Graphical map of the Williams’s jerboa mitogenome. Genes outside the circle were coded on the H-strand, while genes inside the circle were coded on the L-strand. All tRNA genes were denoted by IUPACIUB one-letter amino acid abbreviation. Arrows showed transcription direction of the genes.

The mitogenome assembly of Williams’s jerboa had a mean coverage of 5.800× (min: 1.780, max: 13.432) and a total length of 16,653 bp (NCBI accession number: MT079957). The mitogenome was similar in structure to those of other Dipodoidea mitogenomes (Yue et al., 2015; Ding et al., 2016; Luo & Liao, 2016; Luo, Ding & Liao, 2016; Reyes et al., 2016, https://www.ncbi.nlm.nih.gov/nuccore/AJ416890.1) and included 13 protein-coding (PCGs), 22 transfer RNA (tRNA), two ribosomal RNA (rRNA) genes, a light chain (L) replication origin (O_L_), and a large non-coding control region (D-loop). The *ND6* gene, O_L_ origin and eight tRNA genes (t*RNA*^*Ala*^, *tRNA*^*Asn*^, *tRNA*^*Cys*^, *tRNA*^*Glu*^, *tRNA*^*Gln*^, *tRNA*^*Pro*^, *tRNA*^*Ser*(UCN)^ and *tRNA*^*Tyr*^) were located on the light chain (L chain), while 12 PCG, 14 tRNA and 2 rRNA genes were located on the heavy chain (H chain) (Fig. 1; Table 1). While the *S. williamsi* mitogenome (16,653 bp) was longer compared with the mitogenomes of *S. concolor* (16,492 bp), *J. jaculus* (16,546 bp) and *E. setchuanus* (16,573 bp), it was also shorter relative to the mitogenomes of *D. sagitta* (16,664 bp), *O. sibirica* (16,685 bp), *S. telum* (16,696 bp) and *E. naso* (16,705 bp). The nucleotide composition of the mitogenome was similar to that of typical mammalian mitochondrial genomes, guanine had the lowest frequency (A > T > C > G), with A+T content (59.72%) higher than G+C content (40.28%). The A+T ratio of the D-loop was relatively lower (57.45%) than those of the whole mitogenome (59.72%), PCGs (59.47%), rRNAs (60.40%) and tRNAs (62.88%) (Table S2). Similar to other vertebrate mitogenomes (Ding et al., 2019; Krause et al., 2008; Wada et al., 2010), annotation of the Williams’s jerboa mitogenome showed overlapping regions and intergenic intervals between protein-coding and tRNA genes. The *S. williamsi* mitogenome had nine overlapping regions with a total length of 66 bp (between 1 and 43 bp) and 13 intergenic intervals with a total length of 42 bp (between 1 and 7 bp) (Table 1).

**Table 1:** Annotation of whole mitogenome of Williams’s jerboa.

Gene	Strand	Start position	Lengh (bp)	Intergenic spacer	Start codons	Stop codons	Anticodon
*tRNA-Phe*	L	1–69	69	+3			GAA
*12S rRNA*	L	73–1,036	964	0			
*tRNA-Val*	L	1,037–1,104	68	0			TAC
*16S rRNA*	L	1,105–2,688	1,584	0			
*tRNA-Leu2*	L	2,689–2,763	75	+3			TAA
*ND1*	L	2,767–3,723	957	−2	ATG	TAG	
*tRNA-Ile*	L	3,722–3,790	69	−3			GAT
*tRNA-Gln*	H	3,788–3,858	71	0			TTG
*tRNA-Met*	L	3,859–3,928	70	0			CAT
*ND2*	L	3,929–4,972	1,044	−2	ATA	TAG	
*tRNA-Trp*	L	4,971–5,038	68	+3			TCA
*tRNA-Ala*	H	5042-5112	71	+1			TGC
*tRNA-Asn*	H	5,114–5,187	74	0			GTT
*OL*	H	5,188–5,220	33	0			
*tRNA-Cys*	H	5,221–5,286	66	0			GCA
*tRNA-Tyr*	H	5,287–5,353	67	+1			GTA
*COX1*	L	5,355–6,899	1,545	−3	ATG	TAA	
*tRNA-Ser2*	H	6,897–6,965	69	+5			TGA
*tRNA-Asp*	L	6,971–7,037	67	0			GTC
*COX2*	L	7,038–7,721	684	+4	ATG	TAA	
*tRNA-Lys*	L	7,726–7,793	68	+1			TTT
*ATP8*	L	7,795–7,998	204	−43	ATG	TAA	
*ATP6*	L	7,956–8,636	681	−1	ATG	TAA	
*COX3*	L	8,636–9,419	784	0	ATG	T--	
*tRNA-Gly*	L	9,420–9,487	68	0			TCC
*ND3*	L	9,488–9,835	348	+2	ATA	TAA	
*tRNA-Arg*	L	9,838–9,906	69	+1			TCG
*ND4L*	L	9,908–10,204	297	−7	ATG	TAA	
*ND4*	L	10,198–11,575	1,378	0	ATG	T--	
*tRNA-His*	L	11,576–11,642	67	0			GTG
*tRNA-Ser1*	L	11,643–11,700	58	−1			GCT
*tRNA-Leu1*	L	11.700–11,769	70	0			TAG
*ND5*	L	11,770–13,578	1,809	−4	ATC	TAG	
*ND6*	H	13,575–14,099	525	0	ATG	TAA	
*tRNA-Glu*	H	14,100–14,167	68	+4			TTC
*CYTB*	L	14,172–15,311	1,140	+7	ATG	AGA	
*tRNA-Thr*	L	15,319–15,385	67	+7			TGT
*tRNA-Pro*	H	15,393–15,459	67	0			TGG
**D-loop**	L	15,460–16,653	1,194	0			

**Note:**

IGN, intergenic nucleotide, minus indicates overlapping between genes. tRNAX, where X was the abbreviation of the corresponding amino acid.

**Figure 2:**
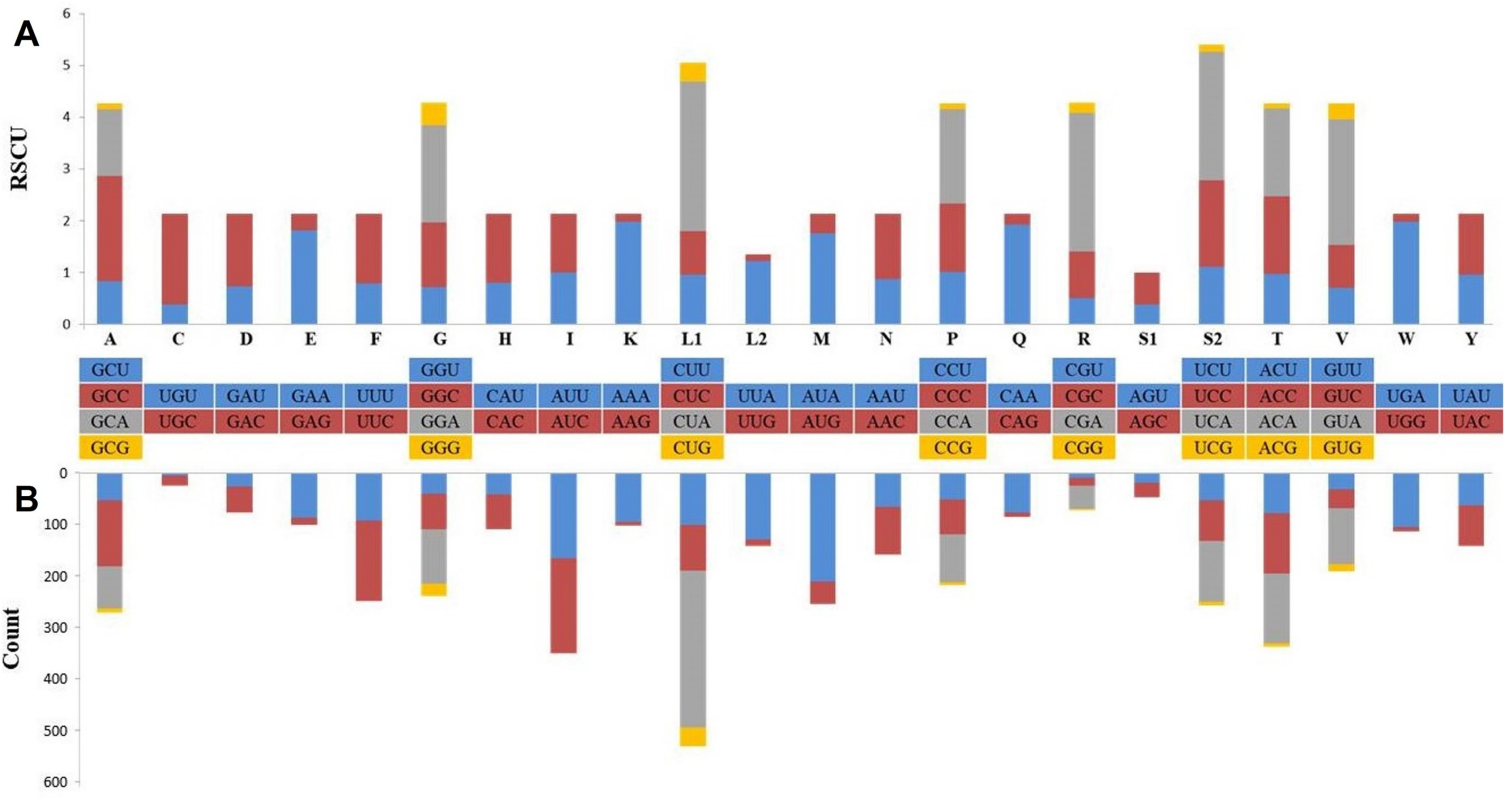
Relative synonymous codon usage (RSCU) (A) and codon composition counts (B) in protein-coding genes of the Williams’s jerboa mitogenome. Amino acids were denoted by IUPACIUB one-letter abbreviation. Codon families were provided on the *X*-axis. The stop codons were not given.

Protein coding genes with a total length of 11,396 bp constituted 68.5% of the mitogenome and coded a total of 3,787 amino acids, except for the termination codons. While the open reading frame of 10 protein-coding genes started with the ATG codon (76.9%), the *ND2* and *ND3* genes started with an ATA codon and the *ND5* gene started with the ATC codon. Eleven of the PCGs were terminated with full termination codons; TAA (*ATP6*, *ATP8*, *COX1*, *COX2*, *ND3*, *ND4L* and *ND6*), TAG for *ND1*, *ND2* and *ND5* genes and AGA for *CYTB*. Moreover, *COX3* and *ND4* were terminated with a T—incomplete termination codon (Table 1). In many metazoan taxa, mitochondrial protein-coding genes may contain an incomplete termination codon (Boore, 1999). This incomplete termination codon can form a complete termination codon through post-transcriptional polyadenylation (Ojala, Montoya & Attardi, 1981). When compared with the available Dipodoidea mitogenomes, the *S. williamsi* mitogenome showed was a deletion of a serine residue at the C-terminus of the *ATP8* gene, similar to observations reported by Yue et al. (2015) for *E. setchuanus* and *S. concolor*. However, this deletion was not observed in the mitogenomes of *D. sagitta*, *J. jaculus* and *E. naso*. None of the remaining PCGs showed evidence of insertions or deletions. While 344 amino acids are encoded by *ND2* in Muridae species and *S. concolor*, 345 amino acids are encoded by *ND2* in Cricetidae species (Yue et al., 2015). By comparison, 347 amino acids are encoded by *ND2* in *S. williamsi* (This Study) and in a great majority of the remaining rodents (Yue et al., 2015). Relative Synonymous Codon Usage (RSCU) and codon usage analyses of the mitogenome are presented in Fig. 2. Among the amino acids that coded on the mitogenome, those with hydrophobic characteristics were the most frequently encoded (*n* = 2,379, 62.6%) while those with acidic groups were the least frequently encoded (*n* = 165, 4.3%). The most frequently used codons were CTA (*n* = 284, 7.5%), ATA (*n* = 196, 5.2%) and ATC (*n* = 172, 4.5%); AGA (*n* = 1), TAG (*n* = 3), CGG (*n* = 3) and TGT (*n* = 4) were rarely used codons. AGG was not used at all.

The lengths of the tRNAs ranged from 58 bp to 75 bp (total length, 1,506 bp; Table 1). The secondary structures of all tRNAs indicated a typical clover shape; however *tRNA*^*Ser(AGY)*^ did not have the typical dihydropyridine (DHU) arm (Fig. S1). The missing clover structure in *tRNA*^*Ser*(AGY)^ could be activated through structural compensation mechanisms between the other arms (Steinberg & Cedergren, 1994). The two rRNA genes had lengths of 964 bp (*12S rRNA*) and 1,584 bp (*16S rRNA*), and were located between *tRNA*^*Phe*^ and *tRNA*^*Leu*(UUR)^; these rRNAs separated from each other by *tRNA*^*Val*^. The putative origin of replication of the light strand (O_L_), one of the non-encoding regions of the mitogenome, was within the WANCY tRNA region. The replication starting sequence at the base of the O_L_ stem (5′-GCCGG-3′), which is commonly found in Dipodoidea members, can show variations among species and genera (Ding et al., 2016) and was determined in the *S. williamsi* mitogenome. The control region (D-loop) of the mitogenome, the largest non-encoding region, was located between *tRNA*^*Pro*^ and *tRNA*^*Phe*^ and was 1,194 bp in length. The D-loop, which includes regions a participating in the replication and transcription of mitogenome, is characterized by separate and conserved sequence blocks and divided into three large groups as the extended termination-associated sequence (ETAS), the central conserved domain, and the conserved sequence block (CBS) (Saccone, Pesole & Sbisa, 1991). The *S. williamsi* D-loop included all three regions (ETAS, 449 bp; central conserved domain, 300 bp; and CSB, 445 bp). It also included twelve 22 bp-long (CATACACACGTACACGCATACG) and one 11 bp-long tandem repeat elements totaling 275 bp in length. A similar pattern, that is, 6–14 bp-long motifs repeated in various numbers was previously observed in the mitogenomes of other Dipodoidea members (Yue et al., 2015; Ding et al., 2016; Luo & Liao, 2016; Luo, Ding & Liao, 2016; Reyes et al., 2016, https://www.ncbi.nlm.nih.gov/nuccore/AJ416890.1). In this context, the longest tandem repeat motif was observed in the *S. williamsi* mitogenome. This finding supports the view that the tandem repeat sequences found in the control regions are common within Dipodinae species (Yue et al., 2015).

### Genetic diversity and phylogenetic analysis

Molecular-based studies on *S. williamsi* samples are scarce and only partial mitochondrial sequences of this species are available in the NCBI database (Dianat et al., 2013; Kryštufek et al., 2013; Hamidi, Darvish & Matin, 2016; Olgun Karacan et al., 2019, https://www.ncbi.nlm.nih.gov/nuccore/MG255335.1). Kryštufek et al. (2013) established the mitochondrial *CYTB* (1,104 bp) and *16S rRNA* (317 bp) sequences of three Williams’s jerboas from Turkey and found that their samples were grouped with samples of *S. williamsi* from north western Iran and samples of *S. euphraticus* from Lebanon. Kryštufek et al. (2013) suggested that the distributional area of *S. williamsi* extends to southern Lebanon, Turkey, and Iran; the authors also mentioned that *S. euphraticus* samples were different from those obtained from Syria and formed a different lineage. Kryštufek et al. (2013) emphasized the presence of a cryptic species within *S. euphraticus*. Olgun Karacan et al. (2019, https://www.ncbi.nlm.nih.gov/nuccore/MG255335.1) recorded the *CYTB* sequences (888 bp) of *S. williamsi* samples in the NCBI database. Besides these studies, two other molecular-based studies (Dianat et al., 2013; Hamidi, Darvish & Matin, 2016) used *S. williamsi* samples from Iran. In these studies, Dianat et al. (2013) used *CYTB* (894 bp) and *COX1* (632 bp) sequences of nine samples from different regions of Iran and suggested that *S. williamsi* is a species complex depending on the genetic variability within the genus. Hamidi, Darvish & Matin (2016) reported the first distributional record of *S. williamsi* (*S. cf. williamsi*) from northeastern Iran, in the Kopet-Dag Mountains based on the *CYTB* (695 bp) sequence obtained from a single sample.

**Figure 3:**
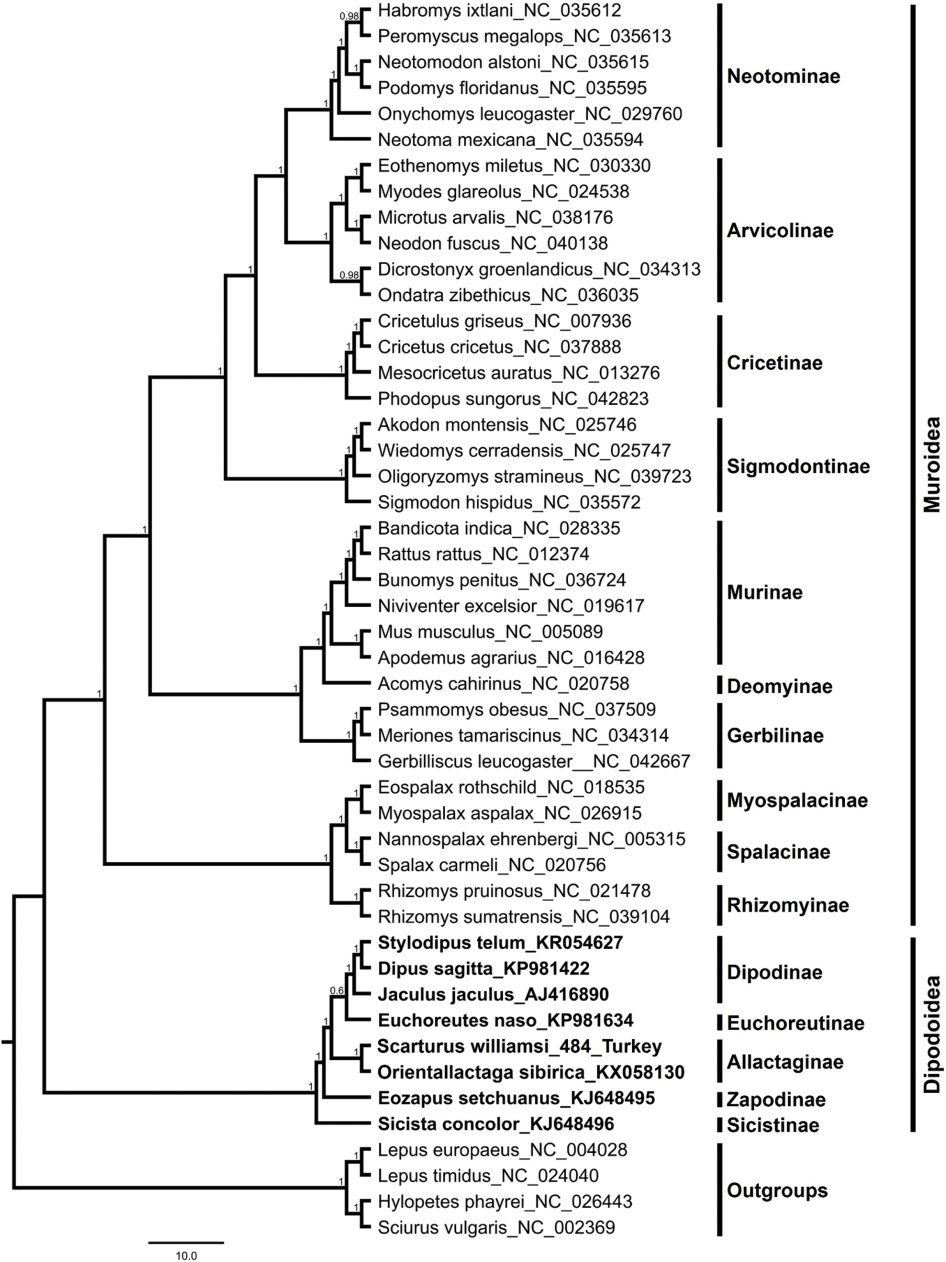
Bayesian tree displaying the phylogenetic relationships between *Scarturus williamsi*, seven other Dipodoidea species and 36 Muroidea species based on mitogenome sequences and GTR+G+I model. Numbers above the branches are Bayesian posterior probabilities (out of 4 million MCMC iterations).

The *S. williamsi* mitogenome obtained in the present study and the mitogenomes of 43 Myomorpha species were combined into a multiple sequence alignment without the D-loop region and analyzed. The genetic distance between the mitogenomes of *S. williamsi* and *O. sibirica* was calculated as 0.176 (17.6%) according to the K2P nucleotide substitution model. The average genetic distance within Dipodinae is 0.178 (17.8%). Mean genetic distances (between-group mean distance) among subfamily Allactaginae and subfamilies Dipodinae, Euchoreutinae, Sicistinae, and Zapodinae were calculated as 0.232 (23.2%), 0.237 (23.7%), 0.304 (30.4%) and 0.268 (26.8%), respectively. BI (Fig. 3) and ML (Fig. S2) analyses produced phylogenetic trees with similar topologies that could be used to delineate subfamilies. All subfamilies within Muroidea formed a well-resolved monophyletic group with high nodal support values. Dipodoidea was located as a basal clade within Myomorpha. The superfamily Dipodoidea formed a sister group with Muroidea, and this relationship has been well demonstrated in previous studies (Holden & Musser, 2005; Steppan, Adkins & Anderson, 2004; Zhang et al., 2013; Michaux & Shenbrot, 2017; Steppan & Schenk, 2017). The results of the present study are in agreement with the results of previous studies. Subfamily Sicistinae, which includes *S. concolor*, formed the most basal branch of Dipodoidea, consistent with previous studies (Blanga-Kanfi et al., 2009; Churakov et al., 2010; Zhang et al., 2013; Yue et al., 2015). *S. williamsi* and *O. sibirica* were found within the subfamily Allactaginae (nodal support: BI, 1.0; ML, 100%), and the species were closely associated with subfamily Zapodinae (*E. setchuanus*) and subfamily Euchoreutinae (*E. naso*). *D. sagitta*, *S. telum*, and *J. jaculus* were clustered together (nodal support: BI, 1.0; ML, 99%) and formed subfamily Dipodinae. The species of subfamily Allactaginae were more basal than the species of Euchoreutinae and Dipodinae (Fig. 3). The results of the current study are largely similar to those of previous studies (Yue et al., 2015; Ding et al., 2016; Luo & Liao, 2016; Luo, Ding & Liao, 2016; Steppan & Schenk, 2017) and support the view that both species should be classified within different genera (*Scarturus* and *Orientallactaga*) (Michaux & Shenbrot, 2017).

## Conclusions

Molecular studies on Williams’s jerboa are scarce, no mitogenome sequence for the species is yet available. This study reports the first mitogenome of Williams’s jerboa as a reference. The mitogenome was 16,653 bp in length; consisted of two rRNAs, 13 PCGs, 22 tRNAs, a control region (D-loop), and an origin of the light-strand replication region (O_L_); and was similar in sequence structure to those of other rodents. The mitogenome included a serine deletion in the C-terminus of the *ATP8* gene, and the *ND2* gene encoded 347 amino acids, similar to the mitogenomes of a great majority of rodents. The mitogenome in the present study included tandem repeat elements in the D-loop region, similar to the mitogenomes of other Dipodoidea species; the longest tandem repeat motif was observed in the Williams’s jerboa mitogenome. According to the phylogenetic trees obtained, superfamily Dipodoidea constituted the most basal group in suborder Myomorpha and superfamilies Dipodoidea and Muroidea were monophyletic and formed a sister group. All of the subfamilies within Muroidea formed a solid monophyletic group, and subfamily Sicistinae was the most basal lineage within Dipodoidea. *S. williamsi* and *O. sibirica* within subfamily Allactaginae may be evaluated as different genera. These results confirm that complete mitogenome sequence are useful in reconstructing the phylogeny of Dipodoidea. However, a great number of taxonomic categories within Dipodoidea remain controversial, and further studies should integrate both morphological and molecular data (mitogenomes, multiloci, autosomal microsatellites and SNPs etc.) of all available taxa to resolve the relationships within this remarkable group of rodents.

## Supplemental Information

10.7717/peerj.9569/supp-1 Supplemental Information 1 The putative secondary structure of 22 tRNA genes found in the Williams’s jerboa mitogenome. All tRNAs were labelled with the abbreviations of their corresponding amino acids.

10.7717/peerj.9569/supp-2 Supplemental Information 2 Maximum likelihood tree displaying the phylogenetic relationships between *Scarturus williamsi*, seven other Dipodoidea species and 36 Muroidea species based on mitogenome sequences and GTR+G+I model.

10.7717/peerj.9569/supp-3 Supplemental Information 3 Primer pairs used for amplification of the Williams’s jerboa mitogenome.

10.7717/peerj.9569/supp-4 Supplemental Information 4 Nucleotide composition of the Williams’s jerboa mitogenome.

10.7717/peerj.9569/supp-5 Supplemental Information 5 Williams Jerboa sequence: MT079957.

## Additional Information and Declarations

### Data Availability

The following information was supplied regarding data availability: The William’s jerboa sequencing data is available at GenBank: MT079957.
