# Supplementary figures and images for: Whole mitochondrial genome sequence and phylogenetic relationships of Williams’s jerboa (Scarturus williamsi) from Turkey
Source: PeerJ. 2020 Jul 16;8:e9569. doi: 10.7717/peerj.9569 (PMC7369027; doi:10.7717/peerj.9569)

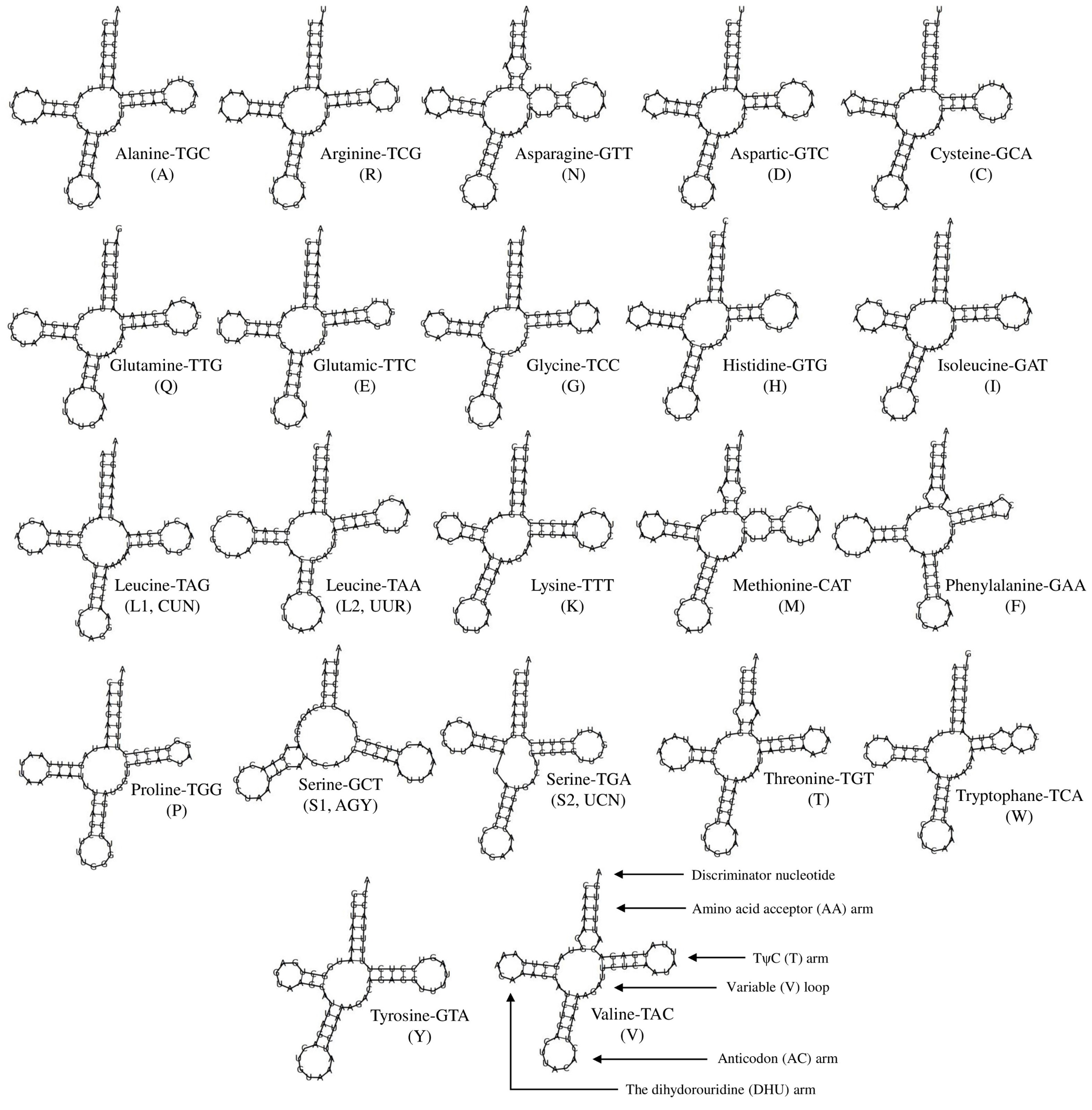

Supplement: Supplemental Information 1 — All tRNAs were labelled with the abbreviations of their corresponding amino acids. [file peerj-08-9569-s001.png]

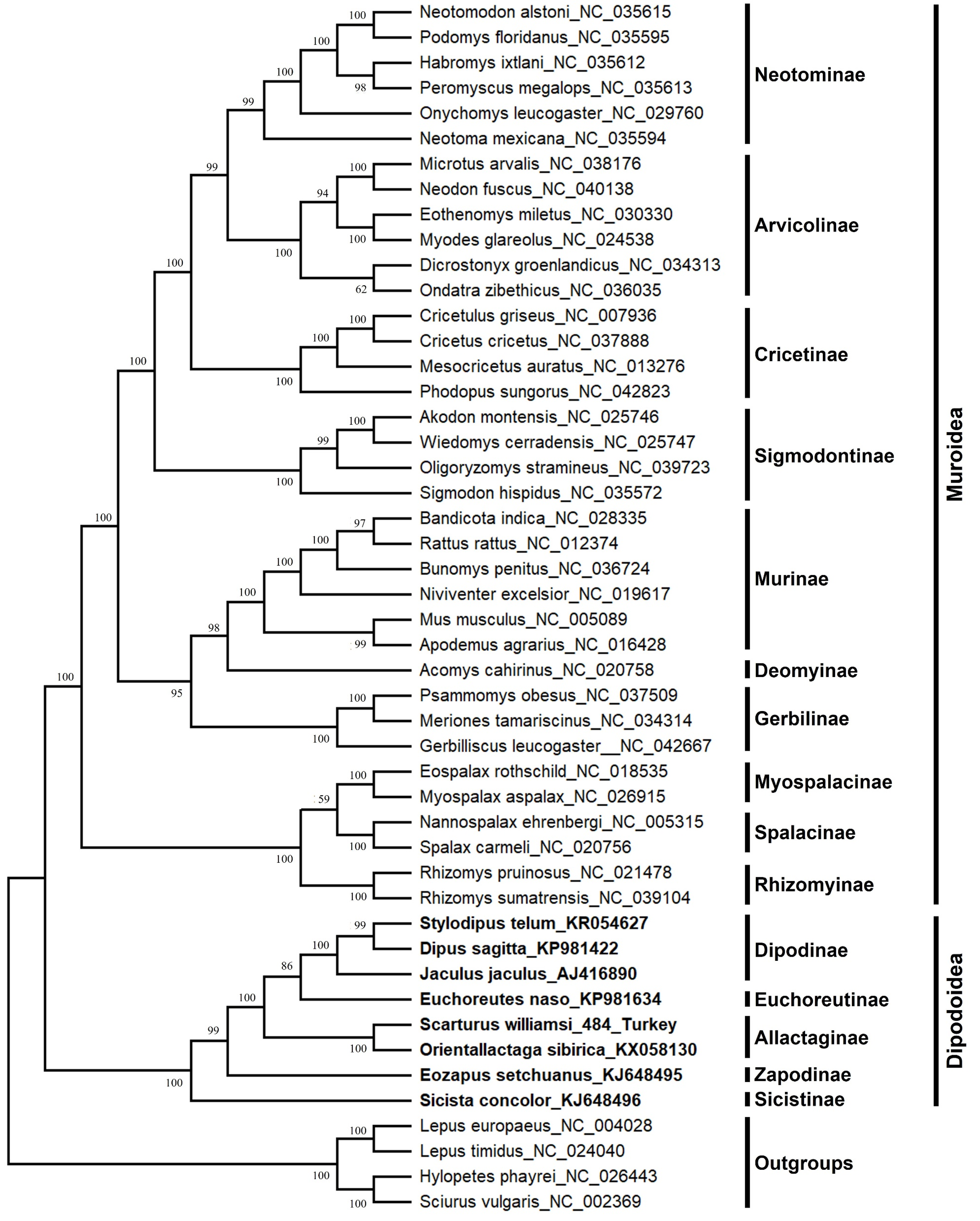

Supplement: Supplemental Information 2 [file peerj-08-9569-s002.png]
