# Supplementary material for: Whole mitochondrial genome sequence and phylogenetic relationships of Williams’s jerboa (Scarturus williamsi) from Turkey
Source: PeerJ. 2020 Jul 16;8:e9569. doi: 10.7717/peerj.9569 (PMC7369027; doi:10.7717/peerj.9569)
Supplement: Supplemental Information 3 [file peerj-08-9569-s003.docx]

**Table S1.** Primer pairs used for amplification of the Williams’s jerboa mitogenome.

| **Primer name** | **Primer sequence (5' --3')** | **Direction** |
| --- | --- | --- |
| CrocAL1_2024L | 5’-GACCGTGCAAAGGTAGCATAATC-3’ | Forward 1 |
| CrocBH1_13002H | 5’- AGAAGTAATCCATTGGTCTTAGGA -3’ | Reverse 1 |
| ScVu-11712L | 5’-TCGGACTAATAATTGTAACCATCG-3’ | Forward 2 |
| LuLu_2503H | 5’-CTCAGATCACGTAGGACTTTAATC-3’ | Reverse 2 |
