# Supplementary material for: Whole mitochondrial genome sequence and phylogenetic relationships of Williams’s jerboa (Scarturus williamsi) from Turkey
Source: PeerJ. 2020 Jul 16;8:e9569. doi: 10.7717/peerj.9569 (PMC7369027; doi:10.7717/peerj.9569)
Supplement: Supplemental Information 4 [file peerj-08-9569-s004.docx]

**Table S2.** Nucleotide composition of the Williams’s jerboa mitogenome.

| Region | Size | % | A(%) | C(%) | G(%) | T(%) | A+T% | G+C% | AT Skew | GC Skew |
| --- | --- | --- | --- | --- | --- | --- | --- | --- | --- | --- |
| **Whole genome** | 16,653 | 100 | 32.55 | 26.93 | 13.35 | 27.17 | 59.72 | 40.28 | 0.09 | -0.34 |
| **PCGs** | 11,396 | 68.5 | 31.56 | 28.75 | 11.78 | 27.90 | 59.47 | 40.53 | 0.06 | -0.42 |
| **rRNA genes** | 2,548 | 15.3 | 35.71 | 21.27 | 18.33 | 24.69 | 60.40 | 39.60 | 0.18 | -0.07 |
| **tRNA genes** | 1,506 | 9.0 | 34.93 | 21.45 | 15.67 | 27.95 | 62.88 | 37.12 | 0.11 | -0.16 |
| **D-loop** | 1,194 | 7.2 | 32.33 | 27.89 | 14.66 | 25.13 | 57.45 | 42.55 | 0.13 | -0.31 |
